# Touring Ensembl: A practical guide to genome browsing

**DOI:** 10.1186/1471-2164-11-295

**Published:** 2010-05-11

**Authors:** Giulietta M Spudich, Xosé M Fernández-Suárez

**Affiliations:** 1 European Bioinformatics Institute, Wellcome Trust Genome Campus, Hinxton, Cambs, CB10 1SD, UK

## Abstract

The number of databases in molecular biological fields has rapidly increased to provide a large-scale resource. Though valuable information is available, data can be difficult to access, compare and integrate due to different formats and presentations of web interfaces. This paper offers a practical guide to the integration of gene, comparative genomic, and functional genomics data using the Ensembl website at http://www.ensembl.org.

The Ensembl genome browser and underlying databases focus on chordate organisms. More species such as plants and microorganisms can be investigated using our sister browser at http://www.ensemblgenomes.org.

In this study, four examples are used that sample many pages and features of the Ensembl browser. We focus on comparative studies across over 50 mostly chordate organisms, variations linked to disease, functional genomics, and access of external information housed in databases outside the Ensembl project. Researchers will learn how to go beyond simply exporting one gene sequence, and explore how a genome browser can integrate data from various sources and databases to build a full and comprehensive biological picture.

## Background

The ongoing increase in the number of databases in biological fields provides a large-scale resource. Last year saw the development of nearly 100 new molecular biological databases, bringing the total number of popular databases in this field to over 1,000 [1]. However, different formats and presentations of the GUIs (graphical user interfaces) make it difficult to access data. Collecting biological information from various sources and comparing them can be time consuming for the researcher. Genome browsers provide an aid to the researcher by importing biological data from various sources and presenting these data in an integrated way.

Three multi-species genome browsers are widely used by the scientific community: the UCSC genome browser, NCBI Map Viewer, and Ensembl (Table 1). Others include H-INvDB (for human) or the FlyBase genome browser, and focus on one or a few species. These browsers not only display information, they tie together annotation from various sources and present it in an integrated way to simplify the view of features along a genome. This article focuses on the power of using a genome browser to go beyond simple questions like 'where are histone modification sites found in the genome' to a more integrated query such as 'where do regulatory features and conserved regions match up in the 5'UTR of a gene.' This allows a more hypothesis-building approach to determining new and undiscovered regions of the genome that may confer function. Genome browsers can be used to deduce function of novel proteins through association with other genes across species. Scientists can use these data to support findings, or to make new hypotheses to be tested through experimentation. The aim of this article is to show how information annotated and presented in genome browsers can enhance hypothesis-driven research.

**Table 1:** Genomics Resources

Name	Website	Function
Genome Browsing Ensembl	http://www.ensembl.org	Genome browser and annotation *(chordates)*
Ensembl Genomes	http://www.ensemblgenomes.org	Genome browser and annotation *(nonchordates)*
Gbrowse	http://flybase.net/cgi-bin/gbrowse/dmel/	Genome browser and annotation (*fruit fly)*
NCBI Map Viewer	http://www.ncbi.nlm.nih.gov/mapview/	Genome browser and annotation *(multi-species)*
NCBI Sequence Viewer	http://www.ncbi.nlm.nih.gov	Genome browser and annotation *(multi-species)*
UCSC Browser	http://genome.ucsc.edu/	Genome browser and annotation *(multi-species)*
VISTA Enhancer Browser	http://enhancer.lbl.gov/	Non-coding elements *(human)*
1000 Genomes Browser	http://browser.1000genomes.org/	Genome browser *(human, multiple individuals)*
IGB	http://tinyurl.com/n83lnn	Visualization tool *(multiple sources)*
IGV	http://www.broadinstitute.org/igv/	Visualization tool *(multiple sources)*
DAS DAS Registry	http://www.dasregistry.org/	DAS sources available
Ensembl DAS list	http://www.ensembl.org/das/dsn	DAS sources available in Ensembl
Protein and Nucleotides RefSeq	http://www.ncbi.nlm.nih.gov/RefSeq	Repository of nucleic acid and protein sequences
UniProt	http://www.uniprot.org	Repository of protein sequence (manually curated)
Tools BioMart	http://www.biomart.org	Data mining tool for export of tables and sequences
EMBOSS	http://emboss.sourceforge.net/	Open source software for molecular biology
TreeBest	http://treesoft.sourceforge.net/njtree.shtml	Construction and analysis of phylogenetic trees
Regulatory Features CisRed	http://www.cisred.org	Database: Regulatory sequences
DNase I Footprint	http://www.flyreg.org	Database: Transcription factor binding sites (*fly)*
miRanda	http://www.microrna.org	Database: miRNA targets *(multi-species)*

Major genomics resources with links to websites.

We focus on the Ensembl genome browser in this article, though a similar approach can be used with other genome browsers shown in table 1. The Ensembl project focuses on the chordate genomes, with the inclusion of additional model organisms that have been extensively studied in biological research and have a reliable, manually annotated gene set (*Caenorhabditis elegans, Drosophila melanogaster *and *Saccharomyces cerevisiae*). In addition to providing carefully predicted gene sets based on experimental evidence (sequences from UniProtKB/Swiss-Prot [2], manually-curated sequences from NCBI RefSeq [3], and sequences from UniProtKB/TrEMBL (Table 1)), Ensembl includes annotation such as sequence variation, comparative associations, mRNA and protein from other databases, predicted features such as CpG islands [4], and repeats and motifs mapped along the genome. These annotations are graphically depicted along the genomic assembly in order to allow easier visualisation of a gene neighbourhood or a stretch of sequence.

Ensembl and other browsers provide displays of complex data sets that require time and computing power not generally available to the researcher. Homology relationships based on gene comparisons across all annotated species in Ensembl (53 species in release 55), along with whole-genome alignments, such as alignments of 31 mammalian genomes, can be readily viewed in the browser.

## Case Studies

In the following four case studies, we use the Ensembl genome browser to demonstrate how to view and predict functional regions in the genome based on existing evidence. First, we examine known regulatory features for the human *IL2 *gene and discuss how to display these features in Ensembl. These promoter and enhancer-related elements can be readily exported using the BioMart tool [5-7].

In study 2, we use human *MYO6*, a case in which gene regulation is not well-understood. Using comparative genomics, we show how the location of functional sequences may be predicted. In case study 3, we demonstrate how the information in Ensembl can be extended through DAS (the Distributed Annotation System)[8] to view data from external sources. Finally, in study 4, we explore a variation associated with disease phenotypes.

These case studies aim to show how data from different sources can be viewed and compared for a gene or region in Ensembl. For a walk-through of how to use the browser to view comparative genomics, variations, and other Ensembl resources, please see our videos[9] and previous publications [10,11]

## Case Study 1: Regulatory Regions for the IL2 Gene

We investigate *IL2*, the *interleukin 2 *gene, in human (ENSG00000109471). Gene regulation has been studied at the 5' end of the IL2 transcript and flanking sequence [12-14]. Within only 200 bp upstream of the translational start site, binding sites for proteins such as NF-κB, AP-1, and NFAT (nuclear factor of activated T-cells), DNase I hypersensitive sites and a TATA box can all be found. These regions have been shown to be involved in the control of T-cell mediated immune response[15,16].

**Figure 1:**
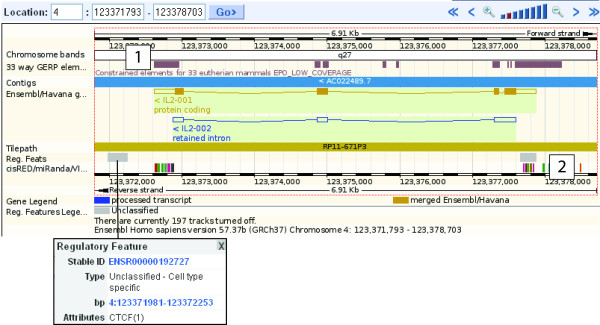
**Conserved Regulatory Regions for IL2**. The 'Region in Detail' view for human genomic assembly GRCh37 [53], chromosome 4, from base pair 123371793 to 123378703 [54], with the IL2 transcript in gold. Boxes show exons, and filled boxes show coding sequence. The direction of translation is indicated by the "<" next to the gene name, and the IL2 transcript is drawn under the blue bar (genome) showing it is on the reverse strand of the chromosome. The constrained elements (label 1) are blocks of highly conserved base pairs resulting from whole genome sequence alignments across 33 species. Regulatory regions from the cisRED and miRanda databases are indicated as coloured, vertical lines (label 2), and match well to the 5'UTR and 5' flank of the IL2 transcript. Regions such as histone modification, DNA methylation, CTCF binding, and DNase I hypersensitive sites are shown as grey shaded boxes (for example, above and left of label 2). Clicking on a box will generate a pop-up window with more information (as shown).

The ENCODE pilot study [17]mapped promoter regions and regulatory sequences in 1% of the human genome, and this approach is now being extended to the entire genome. Ensembl has made a first attempt at annotating these sequences genome-wide by producing a 'regulatory build' based on data from ChIP-Chip[18] and ChIP-Seq [19] experiments (chromatin immunoprecipitation followed by microarray analysis or sequencing, respectively). The ensuing data in the 'Regulatory regions' track in Ensembl are for specific cell types, and include DNase I Hypersensitive sites, CCCTC-binding factor (CTCF) sites, and Histone modification sites (including methylation, acetylation, and alternate histone use)[20]. The IL2 gene possesses features from the regulatory build on the flanking regions to the IL2 transcript (Figure 1).

Pop-up windows reveal more information for each track if a feature is clicked. In figure 1, the pop-up window indicates a CTCF binding site in the regulatory features track. CTCF proteins are highly conserved zinc finger proteins associated with transcriptional activation and repression. Mutations in these genes are associated with invasive breast cancers, prostrate cancers and Wilms' tumours[21,22]. These sites have been recently and extensively mapped onto the human genome[23] and are included in Ensembl as part of the regulatory build.

Regulatory features can be exported using the BioMart tool, or accessed via the Perl API from the Ensembl functional genomics database. An walk-through of the BioMart web interface [24] is provided by Smedley et. al. [5-7] Based on this, to download regulatory features, choose the database as "Ensembl functional genomics" and the dataset as the species of interest. Filters can be applied to select by a region (for example chromosome) or a specific type of regulatory feature (such as DNase I hypersensitive site). Attributes output information (such as chromosomal coordinates, or cell type) about these specific features. For more information about feature sources, and the Ensembl regulatory build, see Ensembl documentation [25].

The "constrained elements" blocks (Figure 1, label 1) are genomic regions that are highly conserved across 33 species, in this example. Constrained elements result from GERP-scoring[26] of each base pair position within a multi-species alignment. High GERP scores represent the most conserved base pairs, and correspond to blocks in the 'conservation' track. The constrained elements in figure 1 align to the 5' and 3' ends of the Ensembl transcript for IL2, and align with regulatory regions, indicating regions of high sequence conservation and thus, possible function.

A third track displays data from 'CisRED'[27] a database of patterns and motifs associated with regulatory regions, 'miRanda'[28] a collection of miRNA targets identified in the genome, and the 'VISTA' enhancer set [29] (Figure 1, **label 2**) Features in this track align to the flanking regions to the IL2 coding sequence, and to the conserved sequence blocks.

**Figure 2:**
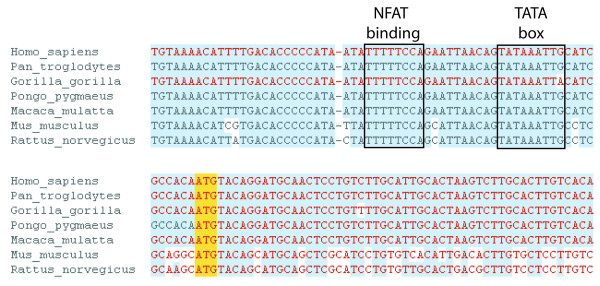
**Conserved Sequences for IL2**. The sequence alignment between mutiple eutherian mammals is shown (*Homo sapiens *(human), *Pan troglodytes *(chimpanzee), *Gorilla gorilla *(gorilla), *Pongo pygmaeus *(orang-utan), *Macaca mulatta *(macaque), *Mus musculus *(mouse), and *Rattus norvegicus *(rat)) [55]. Identical residues are shaded in blue, and the translation start for *IL2 *is shown by a yellow, highlighted box (ATG). Exons are indicated by red sequence. A highly conserved TATA box [56]and NFAT binding site [57] are identified by the authors, and indicated in the figure (i.e. Ensembl does not identify these). The view is accessible through the *genomic alignments *link at the left of the gene or location tabs.

To look more closely at the nucleotide sequence itself, we can view an alignment of the upstream region of the IL2 gene across mammals at the base pair level (Figure 2). To reach this page, click on 'Genomic alignments' at the left of a gene or location page. The sequence in this region is highly conserved across the eutherian mammals shown. The presence of the NFAT (nuclear factor of activated T cells) binding site and TATA box (in the promoter region) for the *IL2 *gene are boxed, along with the translational start site (ATG). This is to illustrate how to view conserved regions in a sequence, and how rich the 5' sequence and flank can be in terms of binding sites and regulatory elements.

The alignment display is highly customisable. Numbering can be turned on or off, and exons highlighted. Pairwise comparisons or multiple alignments can be displayed at the nucleotide level. Alignments can be exported using the *export data *link at the left of the view.

## Case Study 2: Function for a Gene

In case 1, we investigated a gene for which there is information already known about promoter and enhancer elements. Although most human genes in Ensembl are labelled as 'known', signifying a good match to a cDNA or protein in a biological database such as UniProt or NCBI RefSeq, many of these genes have un-investigated regulatory sequences. In addition, many proteins have unknown function. How can we predict function for a protein that is not well-understood in terms of its role in the cell?

In this example we consider human *MYO6*, ENSG00000196586, which has been studied in the mouse model to understand its role in endocytosis and inner-ear development[30,31]. What is known about this gene? We can first look for mouse homologues for the human *MYO6 *gene ENSG00000196586. Do so by clicking on the *orthologues *link at the left of the gene tab for ENSG0000196586. At the time of writing, one mouse orthologue is known for human *MYO6 *(in Ensembl release 55): ENSMUSG00000033577[32].

**Figure 3:**
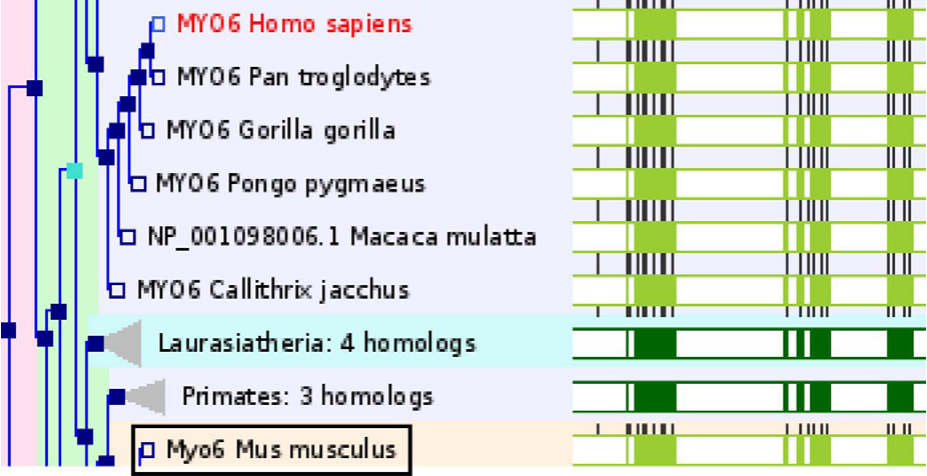
**Gene Tree for Myosin 6**. Protein relationships are clustered into a tree diagram with clickable nodes depicting taxonomic clades, evolutionary events, and links to protein alignments using JalView[34,58]. Red nodes correspond to duplication events, dark blue nodes show speciation events, and light blue nodes are ambiguous duplications. The filled green rectangles at the right demonstrate protein alignments. Light green alignments represent one protein, dark green shading shows a consensus alignment for a collapsed node in the tree. Black ticks in the green bars show positions of introns. Gaps introduced in the alignments are white. Background colouring corresponds to clades, and can be switched off. The tree in the figure shows the human MYO6 protein in red. The mouse orthologue is boxed.

Orthologues and paralogues in Ensembl are determined using phylogenetic gene trees[33]across all available species (Figure 3). In these analyses, scores from blast reciprocal hits are used to cluster proteins in Ensembl for all species. The tree is built from high-scoring clusters. Paralogues result from gene duplications, which are the red nodes in the tree. Orthologues result from speciation events (blue nodes). Nodes in the tree diagram can be clicked on for a duplication confidence score (red nodes), the taxonomic group, and the protein alignments within that branch (viewable through JalView [34]).

**Figure 4:**
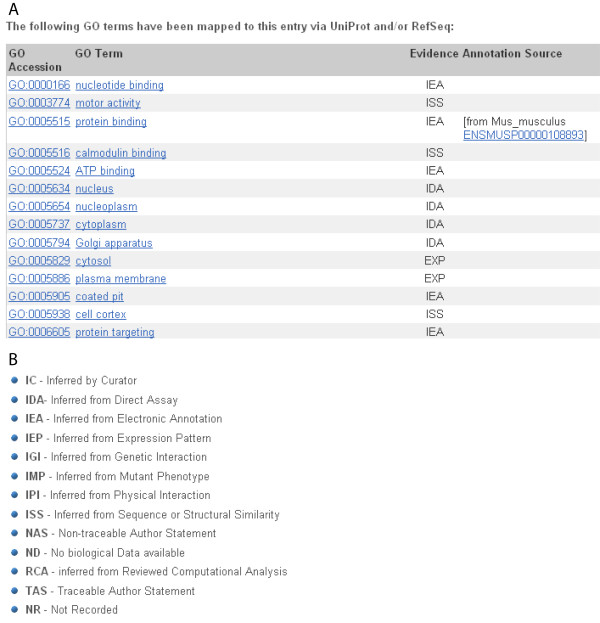
**Gene Ontology for Myosin 6**. Fig 4A. GO terms listed for *Homo sapiens MYO6 *transcript ENST00000428345. Classifications inferred by comparison to the mouse homologue have evidence code IEA. **B**. Description of evidence codes showing how a transcript was assigned to a GO term. More detailed description of these assignments can be found on the Gene Ontology project website [59].

The myosin 6 gene has been extensively studied in the mouse. One way to quickly look for functions associated with the myosin 6 gene is to observe the 'GO terms'[35,36]. These terms are functional classifications designated by the Gene Ontology project[37]. Classifications can be general (e.g. term GO:0005515 protein binding) or more specific (e.g. term GO:0014047 glutamate secretion). GO terms are assigned either by manual curation or an electronic, gene-matching method. The GO terms can be accessed through the transcript tab (the *gene ontology *link at the left). Terms for one human myosin 6 transcript (ENST00000428345) are shown (Figure 4A). The method of GO term assignment is described by a three-letter code. View this 'evidence code' next to the GO term (click 'Help' or visit the GO website to read more about the associations.) (Figure 4B) [38].

Many GO terms for the human *MYO6 *transcript have been projected from mouse homologues (one example is shown in figure 4A). Clicking on the mouse protein identifier ENSMUSP00000108893, then on the *Gene ontology *link at the left shows the GO terms associated to the mouse protein. Protein binding is 'inferred from physical interaction (IPI)' in transcript ENSMUST00000113268[39].

The same GO term is listed for the human *MYO6 *gene in figure 4A, based on homology to the mouse *Myo6 *gene. The evidence code 'IEA' or 'inferred from electronic annotation' demonstrates a projected GO term. They may aid in predicting functions for a protein, based on homology.

Identifying sequences involved in gene regulation is also important in understanding function. In case 1 we looked at the region upstream of the *IL2 *gene, which is rich with known regulatory regions. For the human *MYO6 *gene, we can make some predictions using a similar approach to case 1.

**Figure 5:**
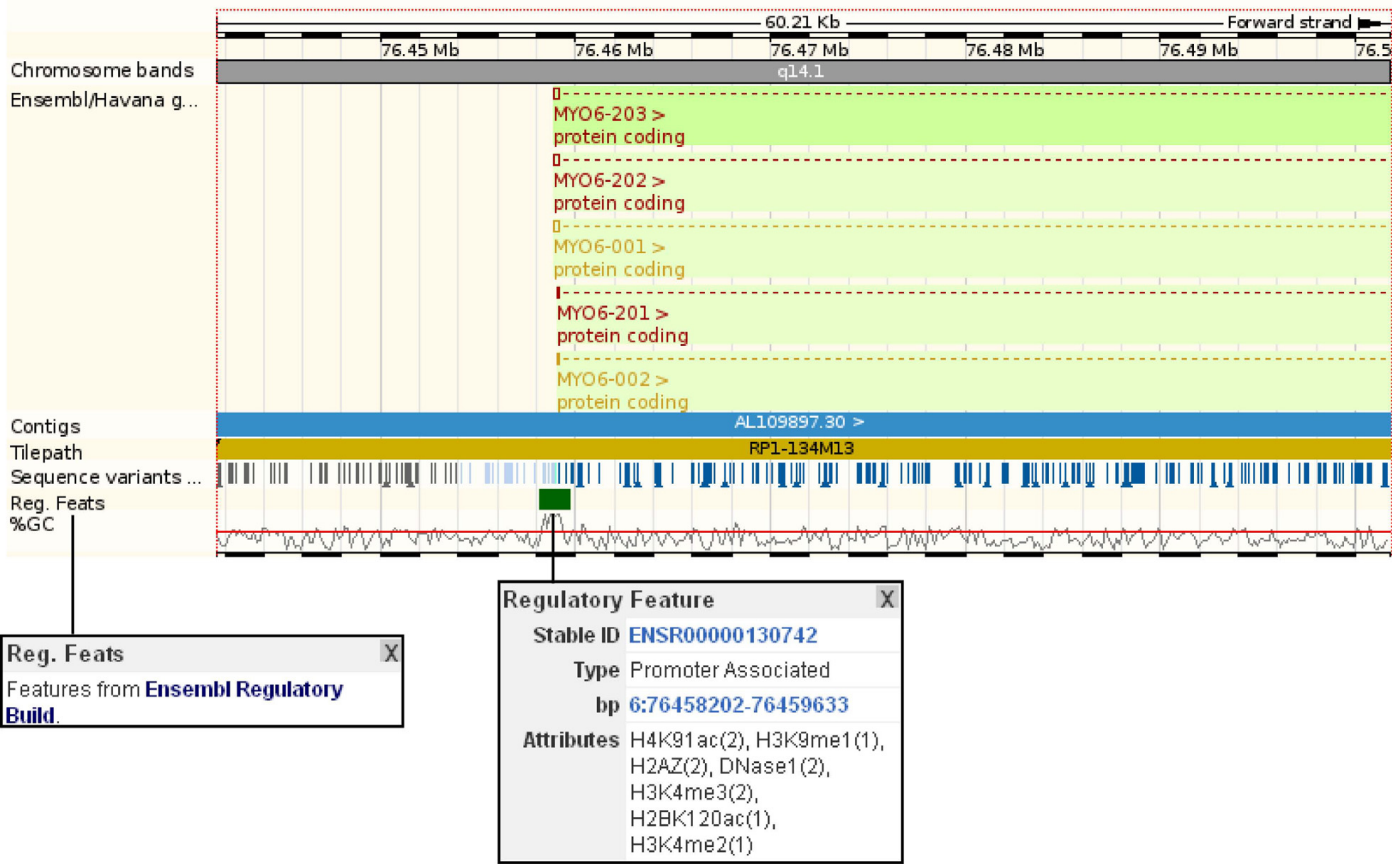
**Predicted Regulatory Features for Myosin 6**. The upstream region and first exon for five myosin 6 transcripts are shown in 'Region in Detail' [60]. Regulatory features are drawn alongside the human genome along with *MYO6 *transcripts, which are on the forward strand. Variations are also drawn and colour-coded indicating the position of the variation with respect to the gene (ie. intronic, upstream, coding). Clicking on variations opens a pop-up box with specific information, and a link to the variation tab. The region shown is chromosome 6, base pairs 76441551 to 76501760. Click on track names or annotation to reveal more information. In the diagram, the information boxes correspond to the regulatory features track.

The 'regulatory features' track in the 'region in detail' view reveal DNase I hypersensitive sites and numerous histone modification and methylation sites aligning to the 5'UTR and upstream region of *MYO6 *transcripts (Figure 5: see the pop-up window at the bottom centre of the figure).

The constrained elements track, and CisRED/miRANDA/VISTA features are also selected in this example. These indicate regions that may function in gene regulation.

In addition, more elements associated with regulatory regions can be displayed along the genome in this view. For example, other elements associated with promoters such as CpG islands[4,40,41], or those determined with FirstEF [42] or Eponine[43] can be selected using the *configure this page *option at the left.

Conclusions from this case study can be drawn from the GO term associations and the putative regulatory regions. Proposed functions for the human *MYO6 *gene and protein include actin filament binding and regulation of secretion [39]. These are based on the known functions of the human *MYO6 *gene homologous to mouse *Myo6*, based on the gene tree. Furthermore, the regulatory build indicates signatures of open chromatin such as CTCF binding sites[21], DNase I hypersensitive sites, along with histone modification sites. Open chromatin and histone modification sites at the 5' end of MYO6 transcripts suggest a potential regulatory region (Figure 5). This sequence could be further investigated for promoter activity.

## Case Study 3: Viewing information outside Ensembl databases

The Distributed Annotation System (DAS)[8] allows Ensembl to link out to and display information from external databases in supported formats. DAS transforms Ensembl into a framework where third party annotation can be added and viewed alongside Ensembl annotation. The DAS registry [44] provides a repository of external sources, and makes it easy for users to select these data to be displayed in Ensembl. These data can be viewed in the browser along the genome, or as annotation for a gene or transcript. This powerful system integrates data from databases around the world, and is available for all species.

**Figure 6:**
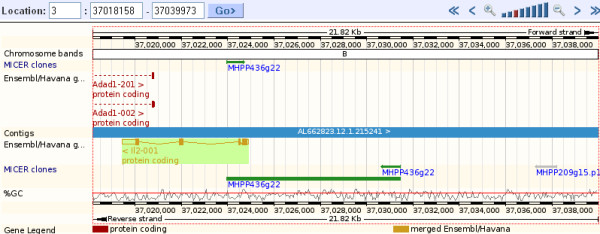
**External MICER Resource**. MICER clones have been selected in the *configure this page *menu for the mouse *IL2 *gene and surrounding regions (NCBIm37 chromosome 3, base pairs 37018158 to 37039973)[61]. The resulting blocks (arrows) represent data from the MICER database, and contain links to MICER information.

Figure 6 demonstrates how to view external data using DAS along the genome. Data from the MICER[45] project (a resource containing vectors and information to generate knock-out mice) is drawn for a region of the mouse genome (Figure 6).

To add DAS tracks to the Location views (Such as Region in Detail, shown in figure 6), users can click on the *configure this page *link at the left. A greater selection of DAS tracks is found upon clicking *manage your data *at the left, and then following the *Attach DAS *link to access the DAS registry. In addition to viewing 'live' external data with DAS, users may draw their own tracks along the chromosome. User data can be displayed in Location views, such as Region in Detail, a chromosome or karyotype[46].

## Case Study 4: From phenotype to SNP- exploring variation

**Figure 7:**
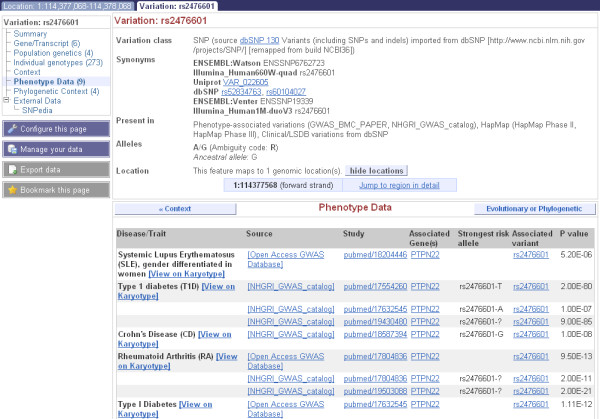
**Variation information for rs2476601**. A search for diabetes in the Ensembl main page shows 16,009 human variations associated with this disease in the NHGRI GWAS catalogue. Searching for one of these, rs2476601, and then clicking on this hit, opens the variation tab for this SNP. The *phenotype data *link at the left ([62]shown in the figure) reveals this variation is implicated in several diseases, including Rheumatoid Arthritis and Crohn's Disease. Links to the GWAS catalogue entries are displayed along with studies in the PubMed database that show the associations. The risk alleles for these diseases are not the same, revealing this position in the genome to be potentially highly important for function.

A new feature in Ensembl is the ability to search with a disease or phenotype. For example, searching for diabetes in the main page results in 350 hits to genes, variations and protein families across species. One of these hits is rs2476601, which Ensembl reveals to have been implicated in Crohn's Disease and Rheumatoid Arthritis, in addition to Type I Diabetes (Figure 7). This information comes from the NHGRI GWAS catalogue [47], and links to publications implicating the variation in the disease and strongest risk alleles can be found in the phenotype data section of Ensembl pages (Figure 7).

Population variation in Ensembl is imported from NCBI dbSNP[48], among other sources[49], and is represented in a variety of views [50]. Clicking on a variation identifier within the Ensembl website opens the variation tab and brings the focus to data for one specific variation, such as a single nucleotide polymorphism (SNP) or insertion-deletion (indel) mutation. Associated data such as allele frequencies from genotype studies done by HapMap [51] or Perlegen[52], or the phenotype information described above can be found in this way.

**Figure 8:**
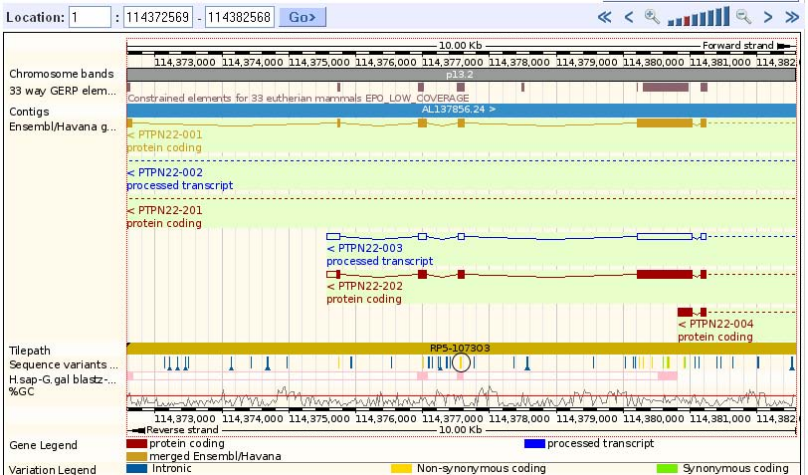
**Region in detail for the rs2476601 locus**. The region around rs2476601[63] is shown, with the non-synonymous SNPs in yellow. rs2476601 is circled, and it aligns with an exon found in two coding, and one non-coding, splice variants of the PTPN22 gene. Note that exons align well with conserved regions, shown by the blocks in the constrained elements track (calculated from the whole genome alignments across 33 species). We can see the deep evolutionary conservation of this region by displaying the human-chicken pairwise alignment (in pink at the bottom of the image), which shows that this region of the genome is likely to be under strong evolutionary constraint throughout vertebrate evolution.

Turning on the variation track in the region in detail page reveals all SNPs, indels, and other variations stored in Ensembl databases and mapped to the position viewed. Position in and effect on the transcript is revealed by the colour of the vertical line signifying the variation. In figure 8, non-synonymous variations (having an effect on the amino acid sequence) are shown as yellow vertical lines. The circled variation is rs2476601, the SNP described above. This variation is within a coding exon in two of the PTPN22 transcripts shown, and has a consequence on the protein sequence in these two splice isoforms. Clicking on the variation reveals a pop-up box showing the ID, the genomic coordinates, and a link to the variation tab shown in figure 7.

## Results and Discussion

Genome browsers have gone beyond the simple display of genes and transcripts, moving into the integration of biological data. Ensembl pages allow information annotated on a genome to be shown alongside genes in one display. This annotation comes from various sources and includes sequence variation, conserved regions, motifs such as CpG islands and sequences associated with regulatory regions and promoters. DAS allows Ensembl to draw together more information in more databases, displaying data from external sources as an added layer of information. It also allows the biological community to display and publish their data in an integrated framework. Furthermore, Ensembl itself is a DAS server, and other browsers may display Ensembl data as a respective external source.

As demonstrated in the case studies outlined here, experimentalists targeting potential functional regions for a gene could use a quick display of a variety of sequence features to form a basis for such predictions. The whole genome alignments leading to comparison of sequences across species can indicate important functional regions that are highly conserved. Regulatory features and associated motifs can be compared with these conserved regions to direct researchers towards undiscovered, potentially functional sites.

## Authors' contributions

Both authors read and approved the manuscript
